# *PPARγ2* Pro12Ala polymorphism was associated with favorable cardiometabolic risk profile in HIV/HCV coinfected patients: a cross-sectional study

**DOI:** 10.1186/s12967-014-0235-9

**Published:** 2014-08-27

**Authors:** Pilar García-Broncano, Juan Berenguer, Amanda Fernández-Rodríguez, Daniel Pineda-Tenor, María Ángeles Jiménez-Sousa, Mónica García–Alvarez, Pilar Miralles, Teresa Aldámiz-Echevarria, Juan Carlos López, Dariela Micheloud, Salvador Resino

**Affiliations:** Unidad de Infección Viral e Inmunidad, Centro Nacional de Microbiología, Instituto de Salud Carlos III, Carretera Majadahonda- Pozuelo, Km 2.2, Majadahonda, Madrid, 28220 Spain; Unidad de Enfermedades Infecciosas/VIH, Hospital General Universitario “Gregorio Marañón”, Madrid, 28007 Spain; Instituto de Investigación Sanitaria Gregorio Marañón (IiSGM), Madrid, 28007 Spain; Servicio de Medicina Interna, Hospital General Universitario “Gregorio Marañón”, Madrid, 28007 Spain

**Keywords:** HIV/HCV coinfection, Serum lipids, Adipokines, Insulin resistance, Fibrosis, Single nucleotide polymorphism

## Abstract

**Background:**

Peroxisome proliferator-activated receptor gamma-2 gene (*PPARγ2*) rs1801282 (Pro12Ala) polymorphism has been associated with lower risk of metabolic disturbance and atherosclerosis. The aim of this study was to analyze the association between the Pro12Ala polymorphism and cardiometabolic risk factors in human immunodeficiency virus (HIV)/Hepatitis C virus (HCV)-coinfected patients.

**Methods:**

We carried out a cross-sectional study on 257 HIV/HCV coinfected patients. *PPARγ2* polymorphism was genotyped by GoldenGate® assay. The main outcome measures were: i) serum lipids (cholesterol, triglycerides, high-density lipoprotein (HDL-C), low-density lipoprotein (LDL-C), LDL-C/HDL-C, and atherogenic index (AI)); ii) homeostatic model assessment (HOMA-IR) values; iii) serum adipokines (leptin, adiponectin, resistin, plasminogen activator inhibitor-1(PAI-1), hepatic growth factor (HGF), and nerve growth factor (NGF)). Generalized Linear Models (GLM) with gamma distribution (log-link) were used to investigate the association between *PPARγ2* polymorphism and continuous outcome variables. This test gives the differences between groups and the arithmetic mean ratio (AMR) in continuous outcome variables between groups.

**Results:**

The rs1801282 CG/GG genotype was associated with low values of cholesterol (adjusted arithmetic mean ratio (aAMR) = 0.87 (95% of confidence interval (95% CI) = 0.79; 0.96); p = 0.004) and LDL-C (aAMR = 0.79 (95% CI = 0.68; 0.93); p = 0.004). Furthermore, rs1801282 CG/GG was associated with low values of HOMA-IR (aAMR = 0.69 (95% CI = 0.49; 0.98); p = 0.038) among patients with significant liver fibrosis (F ≥ 2). Moreover, rs1801282 CG/GG was also associated with low serum values of hepatic growth factor (HGF) (aAMR = 0.61 (95% CI = 0.39; 0.94); p = 0.028), and nerve growth factor (NGF) (aAMR = 0.47 (95% CI = 0.26; 0.84); p = 0.010). The serum levels of leptin, adiponectin, resistin, and PAI-1 did not show significant differences.

**Conclusions:**

The presence of *PPARγ2* rs1801282 G allele (Ala variant) was associated with a protective cardiometabolic risk profile versus CC genotype in HIV/HCV-coinfected patients. Thus, *PPARγ2* rs1801282 polymorphism may play a significant role in the development of metabolic disorders in HIV/HCV coinfected patients, and might have an influence on the cardiovascular risk.

## Background

The introduction of effective combination antiretroviral therapy (cART) has dramatically reduced the mortality and illness related to human immunodeficiency virus (HIV) infection [1]. However, an increased risk of dyslipidemia, insulin resistance, and type 2 diabetes mellitus (T2DM) has been described in human immunodeficiency virus (HIV)-infected individuals who underwent cART [2]. Furthermore, HIV/hepatitis C virus (HCV) coinfection is associated with metabolic disturbance such as dyslipidemia, insulin resistance, and T2DM, which are also associated with an increased risk of cardiovascular disease [3]. These series of factors have increased the risk of cardiovascular diseases in patients with HIV/HCV coinfection in cART era [4,5].

Peroxisome proliferator-activated receptor gamma (PPARγ) is considered as a “key element” in the course of glucose homeostasis, lipoprotein metabolism and vascular homeostasis [6]. The most common polymorphism in *PPARγ2* gene is the Pro12Ala (rs1801282) polymorphism, which generates a cytosine-guanine exchange (CCA-to-GCA missense mutation), affecting the NH2-terminal residue that defines the adipocyte-specific PPARγ2 isoform [7]. The rs1801282 G allele (which encodes Ala) has been associated with lower risk of insulin resistance and T2DM [8,9], hypertension [10], and lower value of carotid artery intima-media thickness, protecting against early atherosclerosis [11–15]. Additionally, rs1801282 polymorphism seems to have an influence on blood lipid levels in different ways, according to the lipid fraction analyzed [16]. However, it has not been found an association between Pro12Ala (rs1801282) polymorphism and metabolic syndrome [17]. In clinical trials with large numbers of patients, the effect of Pro12Ala polymorphism on metabolic disturbances remains controversial since Ala variant has been related to unfavorable changes in serum cholesterol [18], no improvement in lipid variables [19], improvement of plasma lipid levels [20], decreased risk of developing hyperglycemia [21], increased BMI, waist-to-hip ratio, and fasting glucose [22], and weight loss [23]. In the Spanish population, the Ala12 allele was associated with lower total triglycerides levels and increased insulin sensitivity [24].

Probably, Pro12Ala polymorphism may act differently in HIV/HCV coinfected patients due to the fact that HIV proteins and antiretroviral drugs seem to negatively influence the adipocyte *PPARγ* expression [25–28]. Besides, HIV/HCV coinfected patients have an intrinsic metabolic deregulation, which may be enhanced by unhealthy habits that are very pronounced in people coinfected with HIV and HCV (smoking, drug abuse, alcohol, etc.) [29]. Thus, the aim of our study was to analyze the association between the Pro12Ala polymorphism and cardiometabolic risk in HIV/HCV coinfected patients.

## Patients and methods

### Patients

We carried out a cross-sectional study in HIV/HCV-coinfected patients from Hospital Gregorio Marañón (Madrid, Spain) between September 2000 and July 2009. The study was approved by the Institutional Review Board and the Research Ethic Committee (“Comité de Ética de la Investigación y de Bienestar Animal”) of the Instituto de Salud Carlos III. This study was conducted in accordance with the Declaration of Helsinki and patients gave their written consent for the study. All patients were European whites.

All subjects were HCV treatment-naive patients who were potential candidates for HCV therapy and, in most cases, underwent a liver biopsy. The inclusion criteria were: detectable HCV-RNA by polymerase chain reaction, negative hepatitis B surface antigen, availability of DNA sample, no clinical evidence of hepatic decompensation, no diabetes mellitus, and stable cART or no need for cART. Patients with active opportunistic infections, active drug and/or alcohol addiction, and other concomitant diseases were excluded.

A total of 293 HIV/HCV coinfected patients met the inclusion criteria, but only 257 patients were available for analysis: 11 patients were excluded due to genotyping problems and 25 were discarded due to missing data (no homeostatic model assessment (HOMA-IR) data (n = 23) and no lipid data (n = 2)). Besides, only 207 patients had liver biopsy data and 109 patients had an available sample stored at −80°C for testing serum adipokines.

### Epidemiological and clinical data

Medical records were used to obtain epidemiological and clinical data when HCV therapy was started and/or liver biopsy was performed.

The duration of HCV infection for patients with a history of intravenous drug use was estimated starting from the first year they shared needles and other injection paraphernalia, which are the most relevant risk practices for HCV transmission [30]. For non-intravenous drug use patients, we only included those patients for which the initiation of their HCV infection could be determined with certainty.

Biochemistry panel was measured using a fully automated clinical chemistry analyzer (Hitachi 912, Boehringer Mannheim, Germany) in serum of fasting patients. The collected data were total cholesterol (TC), triglycerides (TG) and high-density lipoprotein (HDL-C). The low-density lipoprotein (LDL-C) was calculated by Friedewald estimation (LDL-C = TC – HDL-C – (TG/5)) [31]. The atherogenic risk was estimated for each patient using the atherogenic index (AI) (AI = (TC – HDL-C)/HDL-C). Non HDL-C was calculated as TC minus HDL-C. The degree of IR was estimated for each patient using the HOMA-IR method described by Matthews et al. [32]: fasting glucose (mmol/l) times fasting insulin (mU/l) divided by 22.5.

Liver biopsies were performed as previously described [33]. Liver fibrosis was estimated according to Metavir score. Fibrosis was scored as follows: F0, no fibrosis; F1, portal fibrosis; F2, periportal fibrosis or rare portal-portal septa; F3, fibrous septa with architectural distortion; no obvious cirrhosis (bridging fibrosis); and F4, definite cirrhosis.

### Laboratory assays

Serum adipokines (leptin, adiponectin, resistin, plasminogen activator inhibitor-1 (PAI-1), hepatic growth factor (HGF), and nerve growth factor (NGF)) were measured by multiplex assay using Multiplex kit (LINCOplex™; LINCO Research, St. Charles, Missouri, United States) in the Luminex 100™ analyzer (Luminex Corporation, Austin, Texas, United States), following manufacturer’s specifications.

Genomic DNA was extracted from peripheral blood with Qiagen kit (QIAamp DNA Blood Midi/Maxi; Qiagen, Hilden, Germany). DNA samples were genotyped at the Spanish National Genotyping Center (CeGen; http://www.cegen.org/) for the rs1801282 (C > G) SNP at *PPARγ2* gene. Genotyping was performed by using GoldenGate® assay with VeraCode® Technology (Illumina Inc. San Diego, CA, USA). Moreover, we have also considered other two SNPs that have recently been associated with metabolic disturbances in HIV/HCV coinfected patients: *SLC30A8* rs13266634 [34] and *ADIPOQ* rs2241766 [35]. These SNPs were selected from a review of Staiger et al. about the T2DM risk genes and their genetic variants in patients not infected with HIV and/or HCV [36].

### Outcome variables

i. Dyslipidemia: Serum concentration of TC, TG, HDL-C, LDL-C, LDL-C/HDL-C, and AI.
ii. Insulin resistance: HOMA-IR values.
iii. Serum adipokines: levels of leptin, adiponectin, resistin, PAI-1, HGF, and NGF.

### Statistical analysis

All statistical tests were performed with the Statistical Package for the Social Sciences (SPSS) 19.0 software (IBM Corp., Chicago, USA). All p-values were two-tailed and statistical significance was defined as p < 0.05.

For the description of the study population, p-values were estimated with nonparametric tests: Mann–Whitney U test was used for continuous variable and Chi-square test for categorical variable.

For the genetic association study, the analysis was carried out according to a dominant genetic model of G allele (CC vs. CG/GG), which was the model that best fit to our data. Univariate and multivariate Generalized Linear Models (GLM) with gamma distribution (log-link) were used to investigate the association between *PPARγ2* polymorphism and continuous outcome variables. This test gives the differences between groups and the arithmetic mean ratio (AMR) in continuous outcome variables between groups. All GLM tests were adjusted by the most important clinical and epidemiological characteristics. We included the SNP (Enter algorithm) and the most relevant epidemiological and clinical characteristics (backward criterion with a p-value for exit of 0.20). The covariables used were gender, age, body mass index (BMI), acquired immune deficiency syndrome, nadir CD4+ T-cells, undetectable HIV viral load (<50 copies/mL), time with cART, cART with protease inhibitor, specific antiretroviral drugs (saquinavir, efavirenz, ritonavir, tenofovir, thymidine analogues (AZT, d4T), etc.), HCV genotype, HCV viral load ≥500,000 IU/ml, *SLC30A8* rs13266634 and *ADIPOQ* rs2241766 polymorphisms.

## Results

### Study population

Table 1 shows the main epidemiological and clinical characteristics of the 257 non-diabetic HIV/HCV-coinfected patients (191 men and 66 women).

**Table 1:** **Clinical and epidemiological characteristics of all HIV/HCV coinfected patients stratified by** ***PPARγ2*** **genotype**

		***PPARγ2*** **polymorphism**		
	**All patients (n = 257)**	**CC (n = 223)**	**CG/GG (n = 34)**	**p-value**
**Gender (male)**	191 (74.3%)	165 (74.0%)	26 (76.5%)	0.758
**Age (years)**	40.9 (37.9; 44.7)	41.0 (37.9; 44.7)	40.1 (36.0; 44.7)	0.675
**BMI (kg/m2)**	22.5 (20.9; 24.7)	22.5 (20.9; 24.7)	22.6 (20.7; 24.3)	0.875
**BMI ≥25 kg/m2**	60 (23.4%)	54 (23.9%)	7 (20.6%)	0.674
**HIV acquired by IVDU**	219 (85_.2%)	191 (85.7%)	28 (82.4%)	0.785
**Years since HCV infection**	21.3 (16.6; 24.4)	21.6 (16.7; 24.6)	18.5 (16.1; 23.3)	0.149
**Prior AIDS**	74 (28.8%)	64 (28.7%)	10 (29.4%)	0.973
**cART**	215 (83.7%)	186 (83.4%)	29 (85.3%)	0.831
**Time on cART (years)**	4.8 (2.9; 7.8)	4.7 (2.7; 7.7)	4.9 (3.3; 8.7)	0.490
**Current cART protocols**				
**Any NRTIs + any PI**	62 (24.1%)	51 (22.9%)	11 (32.4%)	0.229
**Any NRTIs + PI + NNRTI**	3 (1.2%)	2 (0.9%)	1 (2.9%)	0.301
**Any NRTIs + any NNRTI**	130 (50.6%)	114 (51.1%)	16 (47.1%)	0.659
**Only NRTIs**	20 (7.8%)	19 (8.5%)	1 (2.9%)	0.258
**Specific antiretroviral drugs**				
**Zidovudine**	71 (7.6%)	63 (28.8%)	8 (23.5%)	0.566
**Stavudine**	66 (25.7%)	55 (25.4%)	11 (32.4%)	0.339
**Didanosine**	41 (16.0%)	35 (15.7%)	6 (17.6%)	0.772
**Tenofovir**	70 (27.2%)	59 (26.5%)	11 (32.4%)	0.472
**Abacavir**	43 (16.7%)	41 (18.4%)	2 (5.9%)	0.069
**Efavirenz**	76 (29.6%)	72 (32.3%)	4 (11.8%)	**0.015**
**Ritonavir**	47 (18.3%)	38 (17.0%)	9 (26.5%)	0.185
**HIV markers**				
**Nadir CD4+ T-cells (cells/μL)**	208 (93; 314.5)	195 (92.5; 306)	257 (145; 364)	0.176
**CD4+ T cells/μL**	467 (341.7; 670.7)	467 (340; 660)	471 (368; 690)	0.678
**HIV-RNA <50 copies/mL**	76.6% (196/256)	77.5% (172/222)	70.6% (24/34)	0.377
**HCV markers**				
**HCV-genotype 1/4**	72.7% (181/249)	72.1% (155/215)	76.5% (26/34)	0.594
**HCV-RNA ≥500,000 UI/ml**	75.6% (186/246)	74.5% (158/212)	82.4% (28/34)	0.324
**Significant fibrosis (F ≥ 2)**	47.8% (99/207)	46.7% (85/182)	56.0% (14/25)	0.383

Categorical variables are expressed in percentage (absolute count). Continuous variables are expressed in median (percentile 25; percentile 75). P-values were estimated with nonparametric Mann–Whitney U test for continuous variable and Chi-square test for categorical variable.

Abbreviations: AIDS acquired immunodeficiency syndrome, BMI body mass index, cART combination antiretroviral therapy, HCV hepatitis C virus, HCV-RNA HCV plasma viral load, HIV human immunodeficiency virus, HIV-RNA HIV plasma viral load, IVDU intravenous drug users, NNRTI no nucleoside analog reverse-transcriptase inhibitors, NRTI nucleoside analog reverse-transcriptase inhibitors, PI protease inhibitors.

The frequency of CG/GG genotype (Ala variant) in our dataset was 13.2%, and was in accordance with minimum allele frequency (MAF) listed on the NCBI SNP database, where the frequency for CG/GG genotypes ranged from 12% to 25% (http://www.ncbi.nlm.nih.gov/projects/SNP/snp_ref.cgi?rs=1801282). *PPARγ2* polymorphism was in Hardy-Weinberg equilibrium (p > 0.05) and displayed less than 5% of missing values.

### *PPARγ2* polymorphism and lipid profile

Patients with rs1801282 CG/GG genotype (Ala variant) had lower values of TC (p = 0.008), LDL-C (p = 0.005), LDL-C/HDL-C ratio (p = 0.027), and AI (p = 0.031) than rs1801282 CC (Pro variant) carriers (Table 2). Furthermore, Ala variant was associated with low values of TC (adjusted AMR (aAMR) = 0.87 ((95% of confidence interval (95% CI) = 0.79; 0.95); p = 0.004) and LDL-C (aAMR = 0.79 (95% CI = 0.68; 0.93); p = 0.004) (Table 2). Moreover, we evaluated the cut-offs for serum lipids (TC ≥ 200 mg/dL, TG ≥ 170 mg/dL, LDL-C ≥ 100 mg/dL, HDL-C ≤ 35 mg/dL, LDL-C/HDL-C ≥ 3.0, and AI ≥ 3.5), but no statistically significant results were found (data not shown).

**Table 2:** **Association between** ***PPARγ2*** **CG/GG genotype (Ala variant) and serum lipids in HIV/HCV coinfected patients**

	**CC**	**CG/GG**	**p-value** ^**(a)**^	**aAMR (95% CI)**	**p-value** ^**(b)**^
TC (mg/dL)	156.0 (63.0)	139.0 (49.0)	**0.008**	0.87 (0.79; 0.95)	**0.004**
TG (mg/dL)	122.0 (49.0)	122.0 (27.0)	0.319	0.92 (0.81; 1.04)	0.163
LDL-C (mg/dL)	86.4 (54.1)	72.4 (36.0)	**0.005**	0.79 (0.68; 0.93)	**0.004**
HDL-C (mg/dL)	36.0 (18.0)	36.0 (12.0)	0.921	0.98 (0.89; 1.09)	0.734
LDL-C/HDL-C	2.1 (1.3)	1.8 (1.1)	**0.027**	0.86 (0.73; 1.01)	0.067
AI	2.7 (1.6)	2.5 (1.4)	**0.031**	0.87 (0.76; 1.01)	0.069

Values expressed as median [interquartile range = upper quartile (Q3) - lower quartile (Q1)] and adjusted arithmetic mean ratio (aAMR) [95% of confidence interval (95% CI)]. Statistically significant differences are shown in bold. ^(a)^P-values were calculated by generalized linear models (GLM) with gamma distribution (log-link). ^(b)^P-values were calculated by GLM with log-link adjusted by the most important clinical and epidemiological characteristics (see Statistical analysis section).

Abbreviations: HCV hepatitis C virus, HIV human immunodeficiency virus, TC total cholesterol, TG triglycerides, LDL-C low density lipoprotein, HDL-C high density lipoprotein, LDL-C/HDL-C ratio of low density lipoprotein/high density lipoprotein, AI atherogenic index.

### *PPARγ2* polymorphism and insulin resistance

Patients with rs1801282 CG/GG genotype had similar HOMA-IR values than patients with rs1801282 CC genotype (Table 3). However, when patients were stratified by liver fibrosis (F < 2 vs. F ≥ 2), rs1801282 CG/GG patients with significant fibrosis (F ≥ 2) had lower HOMA-IR values than rs1801282 CC carriers (p = 0.025) (Table 3). When adjusted GLM analysis was performed among patients with significant liver fibrosis (F ≥ 2), Ala variant was associated with low values of HOMA-IR (aAMR = 0.69 (95% CI = 0.49; 0.98); p = 0.038) (Table 3). Moreover, no statistically significant results were found for the cut-offs of HOMA-IR (≥2.0, ≥2.5, ≥3.0, and ≥3.8) (data not shown).

**Table 3:** **Association between** ***PPARγ2*** **CG/GG genotype (Ala variant) and HOMA-IR in HIV/HCV coinfected patients**

	**CC**	**CG/GG**	**p-value** ^**(a)**^	**aAMR (95% CI)**	**p-value** ^**(b)**^
All patients	2.1 (2.4)	1.7 (2.3)	0.839	0.89 (0.66; 1.20)	0.452
Patients with F < 2	1.8 (1.8)	1.3 (1.5)	0.681	1.01 (0.61; 1.65)	0.988
Patients with F ≥ 2	2.5 (3.3)	2.3 (1.9)	**0.025**	0.69 (0.49; 0.98)	**0.038**

Values expressed as median [interquartile range = upper quartile (Q3) - lower quartile (Q1)] and adjusted arithmetic mean ratio (aAMR) [95% of confidence interval (95% CI)]. Statistically significant differences are shown in bold. ^(a)^P-values were calculated by generalized linear models (GLM) with gamma distribution (log-link). ^(b)^P-values were calculated by GLM with log-link adjusted by the most important clinical and epidemiological characteristics (see Statistical analysis section).

Abbreviations: HCV hepatitis C virus, HIV human immunodeficiency virus, HOMA homeostatic model assessment.

### *PPARγ2* polymorphism and serum adipokine levels

Rs1801282 CG/GG carriers had lower values of HGF (p = 0.003) and NGF (p = 0.008) than rs1801282 CC (Ala variant) carriers (Table 4). When adjusted GLM analysis was performed, Ala variant was associated with low values of HGF (AMR = 0.61 (95% CI = 0.39; 0.94); p = 0.028), and NGF (AMR = 0.47 (95% CI = 0.26; 0.84); p = 0.010) (Table 4). However, we did not find any significant association for adiponectin, leptin, PAI-1, and resistin levels (Table 4).

**Table 4:** **Association between** ***PPARγ2*** **CG/GG genotype (Ala variant) and serum adipokines in HIV/HCV coinfected patients**

	**CC**	**CG/GG**	**p-value** ^**(a)**^	**aAMR (95% CI)**	**p-value** ^**(b)**^
HGF (pg/mL)	1964.9 (2079.2)	1044.4 (1327.1)	**0.003**	0.61 (0.39; 0.94)	**0.028**
NGF (pg/mL)	9.48 (9.43)	6.16 (8.32)	**0.008**	0.47 (0.26; 0.84)	**0.010**
PAI-1 (pg/mL)	1474.9 (17338.2)	1363.1 (7366.9)	0.721	0.783(0.36; 1.69)	0.534
Leptin (pg/mL)	3438.1 (154050.1)	5547.6 (22928.2)	0.529	0.93 (0.55; 1.58)	0.795
Resistin (pg/mL)	1233.1 (15063.9)	1070.4 (2795.7)	0.478	0.76 (0.39; 1.47)	0.425
Adiponectin (ng/mL)	662.9 (10917.3)	1226.6 (4993.1)	0.634	1.14 (0.39; 3.31)	0.800

Values expressed as median [interquartile range = upper quartile (Q3) - lower quartile (Q1)] and adjusted arithmetic mean ratio (aAMR) [95% of confidence interval (95% CI)]. Statistically significant differences are shown in bold. ^(a)^P-values were calculated by generalized linear models (GLM) with gamma distribution (log-link). ^(b)^P-values were calculated by GLM with log-link adjusted by the most important clinical and epidemiological characteristics (see Statistical analysis section).

Abbreviations: HCV hepatitis C virus, HIV human immunodeficiency virus, HGF hepatic growth factor, NGF nerve growth factor, PAI-1 plasminogen activator inhibitor-1.

## Discussion

In this pilot study, rs1801282 G allele (Ala variant) was related to favorable cardiometabolic risk profile with lower levels of serum lipids (TC and LDL-C), HOMA-IR among patients with significant fibrosis (F ≥ 2), and adipokines (HGF and NGF). However, no significant association was found between rs1801282 polymorphism and metabolic disturbance (serum lipids and HOMA-IR values higher than cut-offs).

A recent meta-analysis has shown an inconclusive association between rs1801282 polymorphism and lipid profile in HIV seronegative subjects [16]. This meta-analysis suggests that, compared with rs1801282 CC genotype, carriers of rs1801282 CG/GG genotype have significant increased blood TC, and marginally significant increased blood HDL-C in healthy male subjects [16]. Moreover, there are only three articles published about *PPARγ2* rs1801282 polymorphism in HIV infection [37–39], which did not find any association of rs1801282 polymorphism with lipodystrophy, lipid profile, and insulin sensitivity in HIV-1-infected patients treated with cART [37–39]. In our HIV/HCV coinfected patients, Ala variant carriers had lower values of TC, LDL-C, LDL-C/HDL-C, and AI. In fact, the severe dyslipidemia in these patients may be affected by a large number of factors such as chronic hepatitis C, HIV infection itself, cART used, and body composition changes [29], as well as the traditional factors that increase atherosclerotic risk in the non–HIV-infected population, including genetic factors, diet, alcohol, obesity and inactivity. In our study, it seems clear the protective effect *PPARγ2* Ala variant on serum lipids, being able to have a long-term protective effect on associated cardiovascular disease. In this regard, *PPARγ2* Ala variant has been associated with a reduced risk of hypertension [10], early atherosclerosis [12–15], coronary artery disease [40], and myocardial infarction [41] in general population.

The majority of published data in non HIV-infected patients indicate the association of Ala variant with reduced rates of insulin resistance and T2DM [8,9], although this effect is influenced by genetic heterogeneity [42]. In our study, we only found this favorable effect of Ala variant in patients with significant fibrosis (F ≥ 2), who had the lowest HOMA-IR values. Since approximately 50% of portal insulin is cleared by the liver during first-past transit [43], liver fibrosis might lead to impaired hepatic clearance of insulin and, consequently, it could affect HOMA-IR values. Therefore, rs1801282 polymorphism might play an important role on alterations in insulin metabolism secondary to significant fibrosis. However, the *PPARγ2* rs1801282 polymorphism was not associated with the HOMA-IR cutoffs that indicate IR. This lack of significance might be due to the limited number of patients used in the stratified analysis, or also to the possible distortive effect of direct and indirect factors related to both HIV and HCV infections, and cART [29]. Moreover, we must also take in mind that our patients had a relatively low BMI (22.5 kg/m2), possibly due to the fact that around 85% of our patients were IDUs. HIV infection and chronic drug abuse both compromise nutritional status of patients despite major advances in the HIV treatment [44], allowing that HIV-positive IDUs had lower BMI.

PPARγ is involved in the regulation of adipogenesis, lipid storage, and glucose metabolism [7]. The adipose tissues release a number of adipokines, that may influence on insulin sensitivity and lipid metabolism [45,46], which are regulated directly or indirectly by PPARγ2 [47]. The exact mechanism by which the rs1801282 polymorphism acts is not well understood, but Ala variant at *PPARγ2* gene seems to be an important modulator in metabolic control in the body [7]. In this respect, Ala variant at *PPARγ2* gene seems to reduce transcriptional activity of *PPARγ2* gene, resulting in lower transcription levels of genes activated by *PPAR γ2* and decreasing the process of inflammation and cardiovascular disease [10,12–15]. In addition, the functional effect of rs1801282 evaluated *in silico* has shown that the Alanine variant may affect protein function [48]. Therefore, *PPARγ2* rs1801282 polymorphism could affect the adipokines delivery, since an increase in *PPARγ* gene expression generates the up-regulation of the insulin-sensitizing factor (adiponectin) and down-regulation of insulin-resistant factor (leptin and TNF-*α*) [47], as well as, a less efficient stimulation of PPARγ activated genes predisposing people to lower levels of adipose tissue mass accumulation [49].

Metabolic disorders are closely related to chronic inflammatory response, characterized by abnormal adipokine production, and the activation of proinflammatory signaling pathways, which may play a central role in the cardiovascular pathophysiology [50]. The HGF and NGF are considered adipokines with a possible link to metabolic disorders and other inflammatory-related diseases. HGF and NGF blood levels are elevated in obese subjects and are associated with the presence of metabolic syndrome and T2DM, and both molecules have been described as an inflammatory response protein made by adipocytes [51]. HGF expression, in adipose tissue, is inhibited by NF-κB through suppression of PPARγ function in the HGF gene promoter and PPAR-γ agonists induce HGF expression [52]. In addition, PPAR-γ agonists may also induce NGF expression [53]. In our study, the presence of rs1801282 CG/GG genotype was associated with low serum levels of HGF and NGF, possibly due to the effect of Ala variant on the decreased expression of *PPARγ2* gene, which decrease the process of inflammation and cardiovascular disease [10,12–15]. HGF is a powerful mitogen for hepatocytes and other epithelial tissues mainly produced by perivascular fat cells [54], and plays a special role because it is both a very potent angiogenic growth factor and a cytokine involved in hematopoiesis and vasculogenesis [55,56]. In addition, NGF is a small soluble protein (neurotrophin) that is secreted by various tissues in the body, and it promotes the growth of nerve cell processes and survival of neurons [57]. NGF seems to play a role in several diseases related to cardiovascular risk, such as coronary atherosclerosis, obesity, T2DM, and metabolic syndrome [58].

We have used the same cohort to publish several genetic association studies between SNPs and metabolic disturbances. The SNPs were selected from a review of Staiger et al. about the type 2 diabetes risk genes and their genetic variants in patients not infected with HIV and/or HCV [36], and our approach was to evaluate whether these SNPs also might be markers of metabolic alterations in HIV/HCV coinfected population. Therefore, although we have performed multiple tests, we have not conducted a random search of a meaningful result because these SNPs have been evaluated in general population [36]. Thus, these results should not be affected by adjusting the “p-value” after multiple tests because our study was a clinical-orientated study [59,60].

There are also other issues that have to be considered for a correct interpretation of our data. First, this is a cross-sectional study with a limited number of patients, which could limit achieving statistically significant values. Secondly, metabolic disturbance may be caused by several interacting genetic and environmental determinants, being complicated to find the true individual effects of each disease-associated factor. In this regard, we did not have data on some extra factors that may influence on lipid levels and insulin resistance such as exercise habits, diet, lipodystrophy, and alcohol intake. Thirdly, our study should have been also performed in HCV-monoinfected patients in order to evaluate the significance of only chronic hepatitis C, and in HIV-monoinfected patients in order to evaluate the significance of HIV infection and cART in the development of metabolic disturbances. Fourthly, cART may increase the risk for unfavorable cardiometabolic profile [2]; but in our study, cART characteristics were included in the adjusted regression analyses (see Statistical analysis section). Furthermore, the number of patients taking efavirenz in the rs1801282 CC group was significantly higher than that in the rs1801282 CG/GG group; but we did not find any significant associations among lipid levels and HOMA-IR values with efavirenz (*data not shown*). Fifthly, the patients selected for our study were patients who met a set of criteria for starting HCV treatment and it is possible that this may have introduced a selection bias.

## Conclusions

The presence of rs1801282 G allele (Ala variant) was related to a protective cardiometabolic risk profile in HIV/HCV coinfected patients. Thus, *PPARγ2* rs1801282 polymorphism may play a significant role in the development of metabolic disorders in HIV/HCV coinfected patients, and might have an influence on the cardiovascular risk. However, we consider that further analyses are needed in order to determine the potential use of rs1801282 polymorphism as a marker of cardiovascular risk in HIV/HCV-coinfected patients.

### Meetings at which parts of the data were presented

Some parts have been presented in the 21^th^ Conference on Retroviruses and Opportunistic Infections (CROI): García-Broncano P, Berenguer J, Fernández-Rodríguez A, Pineda-Tenor D, Jiménez-Sousa MA, Cosín J, García–Álvarez M, Miralles P, Aldámiz-Echevarria T, López JC , Micheloud D, Resino S. Pro12Ala Polymorphism Is Associated with Metabolic Disturbance in HIV/HCV Coinfected Patients. 21th Conference on Retroviruses and Opportunistic Infections (CROI 2014). Boston, Massachusetts, USA. March 3–6, 2014.

## Abbreviations

AMR: Arithmetic mean ratio
AI: Atherogenic index
BMI: Body mass index
cART: Combination antiretroviral therapy
HGF: Hepatic growth factor
HCV: Hepatitis C virus
HDL-C: High-density lipoprotein
HOMA-IR: Homeostatic model assessment of insulin resistance
HIV: Human immunodeficiency virus
LDL-C: Low-density lipoprotein
NGF: Nerve growth factor
PAI-1: Plasminogen activator inhibitor-1
PPARγ: Proliferator-activated receptor gamma
TC: Total cholesterol
TG: Triglycerides
T2DM: Type 2 diabetes mellitus
